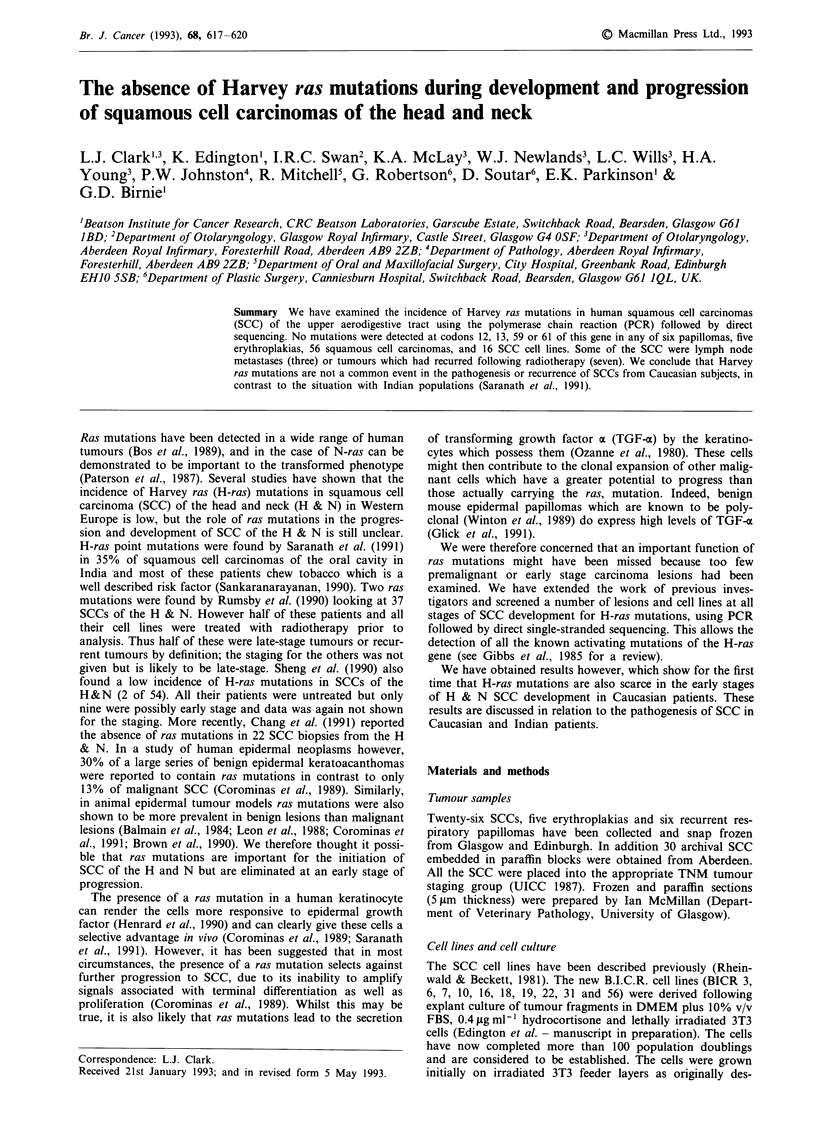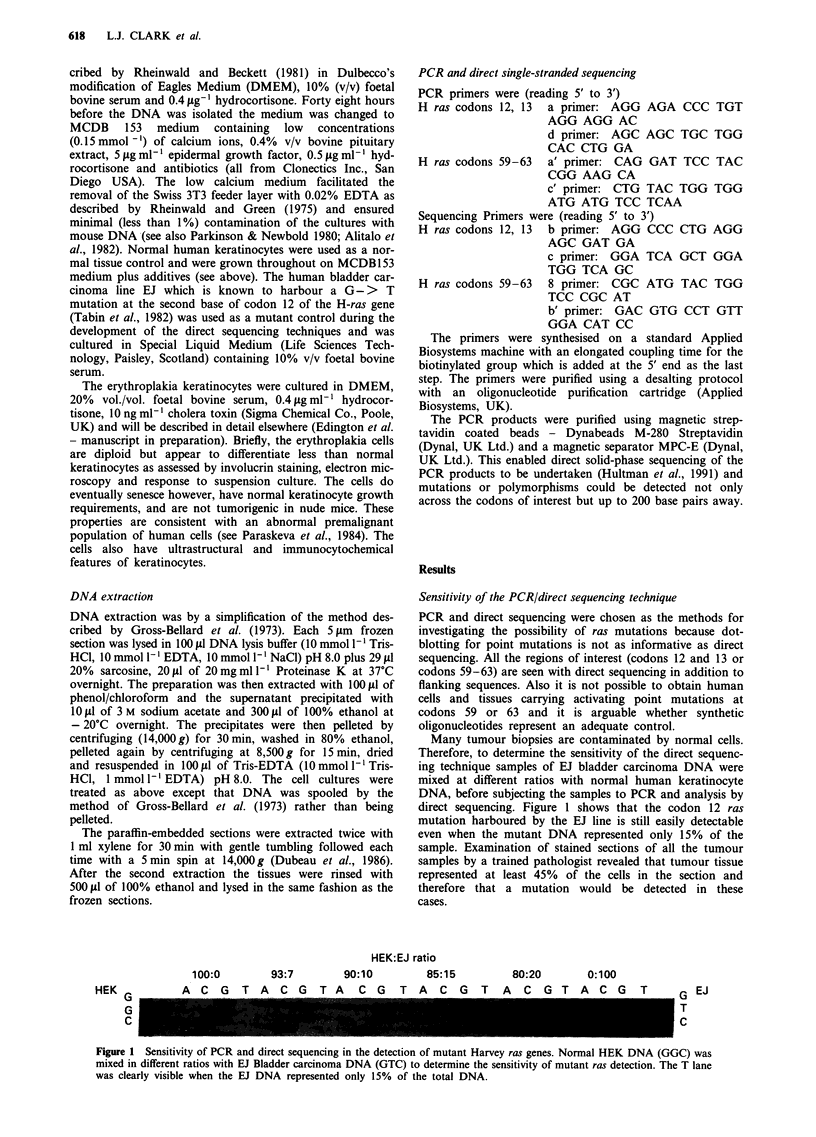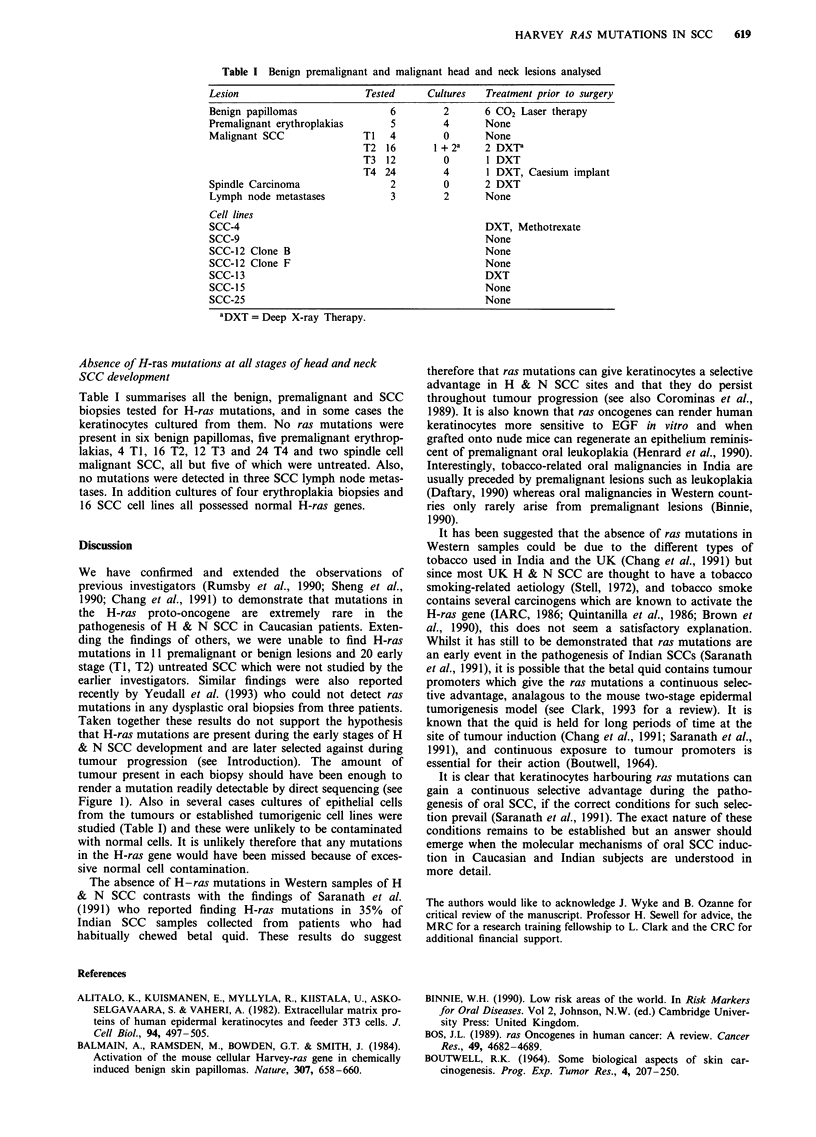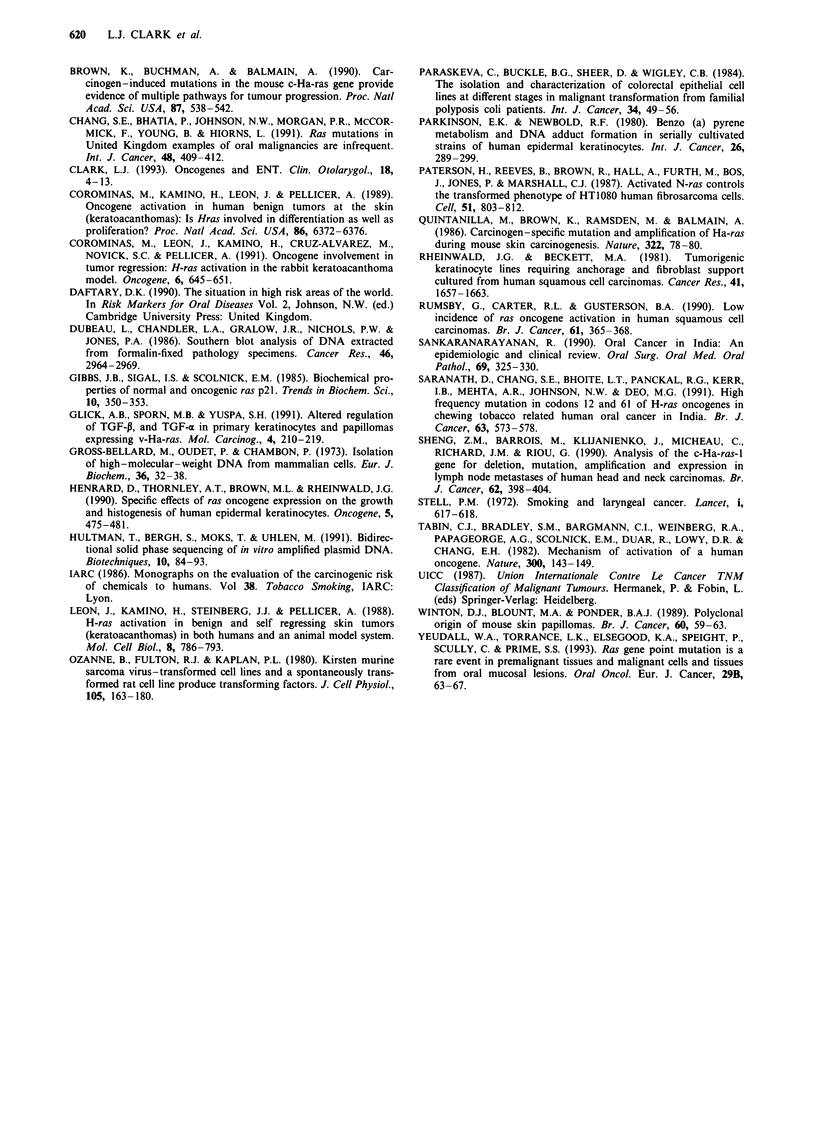# The absence of Harvey ras mutations during development and progression of squamous cell carcinomas of the head and neck.

**DOI:** 10.1038/bjc.1993.396

**Published:** 1993-09

**Authors:** L. J. Clark, K. Edington, I. R. Swan, K. A. McLay, W. J. Newlands, L. C. Wills, H. A. Young, P. W. Johnston, R. Mitchell, G. Robertson

**Affiliations:** Beatson Institute for Cancer Research, CRC Beatson Laboratories, Glasgow, U.K.

## Abstract

**Images:**


*Br. J. Cancer* (1993), **68**, 617–620 © Macmillan Press Ltd., 1993

### The absence of Harvey *ras* mutations during development and progression of squamous cell carcinomas of the head and neck

L.J. Clark[1,3], K. Edington[1], I.R.C. Swan[2], K.A. McLay[3], W.J. Newlands[3], L.C. Wills[3], H.A. Young[3], P.W. Johnston[4], R. Mitchell[5], G. Robertson[6], D. Soutar[6], E.K. Parkinson[1] & G.D. Birnie[1]

*[1]Beatson Institute for Cancer Research, CRC Beatson Laboratories, Garscube Estate, Switchback Road, Bearsden, Glasgow G61 1BD; [2]Department of Otolaryngology, Glasgow Royal Infirmary, Castle Street, Glasgow G4 0SF; [3]Department of Otolaryngology, Aberdeen Royal Infirmary, Foresterhill Road, Aberdeen AB9 2ZB; [4]Department of Pathology, Aberdeen Royal Infirmary, Foresterhill, Aberdeen AB9 2ZB; [5]Department of Oral and Maxillofacial Surgery, City Hospital, Greenbank Road, Edinburgh EH10 5SB; [6]Department of Plastic Surgery, Canniesburn Hospital, Switchback Road, Bearsden, Glasgow G61 1QL, UK.*

**Summary** We have examined the incidence of Harvey *ras* mutations in human squamous cell carcinomas (SCC) of the upper aerodigestive tract using the polymerase chain reaction (PCR) followed by direct sequencing. No mutations were detected at codons 12, 13, 59 or 61 of this gene in any of six papillomas, five erythroplakias, 56 squamous cell carcinomas, and 16 SCC cell lines. Some of the SCC were lymph node metastases (three) or tumours which had recurred following radiotherapy (seven). We conclude that Harvey *ras* mutations are not a common event in the pathogenesis or recurrence of SCCs from Caucasian subjects, in contrast to the situation with Indian populations (Saranath *et al.*, 1991).

*Ras* mutations have been detected in a wide range of human tumours (Bos *et al.*, 1989), and in the case of N-*ras* can be demonstrated to be important to the transformed phenotype (Paterson *et al.*, 1987). Several studies have shown that the incidence of Harvey *ras* (H-*ras*) mutations in squamous cell carcinoma (SCC) of the head and neck (H & N) in Western Europe is low, but the role of *ras* mutations in the progression and development of SCC of the H & N is still unclear. H-*ras* point mutations were found by Saranath *et al.* (1991) in 35% of squamous cell carcinomas of the oral cavity in India and most of these patients chew tobacco which is a well described risk factor (Sankaranarayanan, 1990). Two *ras* mutations were found by Rumsby *et al.* (1990) looking at 37 SCCs of the H & N. However half of these patients and all their cell lines were treated with radiotherapy prior to analysis. Thus half of these were late-stage tumours or recurrent tumours by definition; the staging for the others was not given but is likely to be late-stage. Sheng *et al.* (1990) also found a low incidence of H-*ras* mutations in SCCs of the H&N (2 of 54). All their patients were untreated but only nine were possibly early stage and data was again not shown for the staging. More recently, Chang *et al.* (1991) reported the absence of *ras* mutations in 22 SCC biopsies from the H & N. In a study of human epidermal neoplasms however, 30% of a large series of benign epidermal keratoacanthomas were reported to contain *ras* mutations in contrast to only 13% of malignant SCC (Corominas *et al.*, 1989). Similarly, in animal epidermal tumour models *ras* mutations were also shown to be more prevalent in benign lesions than malignant lesions (Balmain *et al.*, 1984; Leon *et al.*, 1988; Corominas *et al.*, 1991; Brown *et al.*, 1990). We therefore thought it possible that *ras* mutations are important for the initiation of SCC of the H and N but are eliminated at an early stage of progression.

The presence of a *ras* mutation in a human keratinocyte can render the cells more responsive to epidermal growth factor (Henrard *et al.*, 1990) and can clearly give these cells a selective advantage *in vivo* (Corominas *et al.*, 1989; Saranath *et al.*, 1991). However, it has been suggested that in most circumstances, the presence of a *ras* mutation selects against further progression to SCC, due to its inability to amplify signals associated with terminal differentiation as well as proliferation (Corominas *et al.*, 1989). Whilst this may be true, it is also likely that *ras* mutations lead to the secretion of transforming growth factor α (TGF-α) by the keratinocytes which possess them (Ozanne *et al.*, 1980). These cells might then contribute to the clonal expansion of other malignant cells which have a greater potential to progress than those actually carrying the *ras*, mutation. Indeed, benign mouse epidermal papillomas which are known to be polyclonal (Winton *et al.*, 1989) do express high levels of TGF-α (Glick *et al.*, 1991).

We were therefore concerned that an important function of *ras* mutations might have been missed because too few premalignant or early stage carcinoma lesions had been examined. We have extended the work of previous investigators and screened a number of lesions and cell lines at all stages of SCC development for H-*ras* mutations, using PCR followed by direct single-stranded sequencing. This allows the detection of all the known activating mutations of the H-*ras* gene (see Gibbs *et al.*, 1985 for a review).

We have obtained results however, which show for the first time that H-*ras* mutations are also scarce in the early stages of H & N SCC development in Caucasian patients. These results are discussed in relation to the pathogenesis of SCC in Caucasian and Indian patients.

### Materials and methods

### *Tumour samples*

Twenty-six SCCs, five erythroplakias and six recurrent respiratory papillomas have been collected and snap frozen from Glasgow and Edinburgh. In addition 30 archival SCC embedded in paraffin blocks were obtained from Aberdeen. All the SCC were placed into the appropriate TNM tumour staging group (UICC 1987). Frozen and paraffin sections (5 μm thickness) were prepared by Ian McMillan (Department of Veterinary Pathology, University of Glasgow).

### *Cell lines and cell culture*

The SCC cell lines have been described previously (Rheinwald & Beckett, 1981). The new B.I.C.R. cell lines (BICR 3, 6, 7, 10, 16, 18, 19, 22, 31 and 56) were derived following explant culture of tumour fragments in DMEM plus 10% v/v FBS, 0.4 μg $ml^{-1}$ hydrocortisone and lethally irradiated 3T3 cells (Edington *et al.* – manuscript in preparation). The cells have now completed more than 100 population doublings and are considered to be established. The cells were grown initially on irradiated 3T3 feeder layers as originally des-

Correspondence: L.J. Clark.
Received 21st January 1993; and in revised form 5 May 1993.

cribed by Rheinwald and Beckett (1981) in Dulbecco's modification of Eagles Medium (DMEM), 10% (v/v) foetal bovine serum and 0.4 $\mu g^{-1}$ hydrocortisone. Forty eight hours before the DNA was isolated the medium was changed to MCDB 153 medium containing low concentrations (0.15 mmol $^{-1}$) of calcium ions, 0.4% v/v bovine pituitary extract, 5 $\mu g\,ml^{-1}$ epidermal growth factor, 0.5 $\mu g\,ml^{-1}$ hydrocortisone and antibiotics (all from Clonectics Inc., San Diego USA). The low calcium medium facilitated the removal of the Swiss 3T3 feeder layer with 0.02% EDTA as described by Rheinwald and Green (1975) and ensured minimal (less than 1%) contamination of the cultures with mouse DNA (see also Parkinson & Newbold 1980; Alitalo *et al.*, 1982). Normal human keratinocytes were used as a normal tissue control and were grown throughout on MCDB153 medium plus additives (see above). The human bladder carcinoma line EJ which is known to harbour a G–> T mutation at the second base of codon 12 of the H-*ras* gene (Tabin *et al.*, 1982) was used as a mutant control during the development of the direct sequencing techniques and was cultured in Special Liquid Medium (Life Sciences Technology, Paisley, Scotland) containing 10% v/v foetal bovine serum.

The erythroplakia keratinocytes were cultured in DMEM, 20% vol./vol. foetal bovine serum, 0.4 $\mu g\,ml^{-1}$ hydrocortisone, 10 $ng\,ml^{-1}$ cholera toxin (Sigma Chemical Co., Poole, UK) and will be described in detail elsewhere (Edington *et al.* – manuscript in preparation). Briefly, the erythroplakia cells are diploid but appear to differentiate less than normal keratinocytes as assessed by involucrin staining, electron microscopy and response to suspension culture. The cells do eventually senesce however, have normal keratinocyte growth requirements, and are not tumorigenic in nude mice. These properties are consistent with an abnormal premalignant population of human cells (see Paraskeva *et al.*, 1984). The cells also have ultrastructural and immunocytochemical features of keratinocytes.

### *DNA extraction*

DNA extraction was by a simplification of the method described by Gross-Bellard *et al.* (1973). Each 5 $\mu m$ frozen section was lysed in 100 $\mu l$ DNA lysis buffer (10 $mmol\,l^{-1}$ Tris-HCl, 10 $mmol\,l^{-1}$ EDTA, 10 $mmol\,l^{-1}$ NaCl) pH 8.0 plus 29 $\mu l$ 20% sarcosine, 20 $\mu l$ of 20 mg ml $l^{-1}$ Proteinase K at 37°C overnight. The preparation was then extracted with 100 $\mu l$ of phenol/chloroform and the supernatant precipitated with 10 $\mu l$ of 3 M sodium acetate and 300 $\mu l$ of 100% ethanol at – 20°C overnight. The precipitates were then pelleted by centrifuging (14,000 *g*) for 30 min, washed in 80% ethanol, pelleted again by centrifuging at 8,500 *g* for 15 min, dried and resuspended in 100 $\mu l$ of Tris-EDTA (10 $mmol\,l^{-1}$ Tris-HCl, 1 $mmol\,l^{-1}$ EDTA) pH 8.0. The cell cultures were treated as above except that DNA was spooled by the method of Gross-Bellard *et al.* (1973) rather than being pelleted.

The paraffin-embedded sections were extracted twice with 1 ml xylene for 30 min with gentle tumbling followed each time with a 5 min spin at 14,000 *g* (Dubeau *et al.*, 1986). After the second extraction the tissues were rinsed with 500 $\mu l$ of 100% ethanol and lysed in the same fashion as the frozen sections.

### *PCR and direct single-stranded sequencing*

PCR primers were (reading 5′ to 3′)

H *ras* codons 12, 13 a primer: AGG AGA CCC TGT AGG AGG AC
d primer: AGC AGC TGC TGG CAC CTG GA

H *ras* codons 59–63 a′ primer: CAG GAT TCC TAC CGG AAG CA
c′ primer: CTG TAC TGG TGG ATG ATG TCC TCAA

Sequencing Primers were (reading 5′ to 3′)

H *ras* codons 12, 13 b primer: AGG CCC CTG AGG AGC GAT GA
c primer: GGA TCA GCT GGA TGG TCA GC

H *ras* codons 59–63 8 primer: CGC ATG TAC TGG TCC CGC AT
b′ primer: GAC GTG CCT GTT GGA CAT CC

The primers were synthesised on a standard Applied Biosystems machine with an elongated coupling time for the biotinylated group which is added at the 5′ end as the last step. The primers were purified using a desalting protocol with an oligonucleotide purification cartridge (Applied Biosystems, UK).

The PCR products were purified using magnetic streptavidin coated beads – Dynabeads M-280 Streptavidin (Dynal, UK Ltd.) and a magnetic separator MPC-E (Dynal, UK Ltd.). This enabled direct solid-phase sequencing of the PCR products to be undertaken (Hultman *et al.*, 1991) and mutations or polymorphisms could be detected not only across the codons of interest but up to 200 base pairs away.

### Results

### *Sensitivity of the PCR/direct sequencing technique*

PCR and direct sequencing were chosen as the methods for investigating the possibility of *ras* mutations because dot-blotting for point mutations is not as informative as direct sequencing. All the regions of interest (codons 12 and 13 or codons 59–63) are seen with direct sequencing in addition to flanking sequences. Also it is not possible to obtain human cells and tissues carrying activating point mutations at codons 59 or 63 and it is arguable whether synthetic oligonucleotides represent an adequate control.

Many tumour biopsies are contaminated by normal cells. Therefore, to determine the sensitivity of the direct sequencing technique samples of EJ bladder carcinoma DNA were mixed at different ratios with normal human keratinocyte DNA, before subjecting the samples to PCR and analysis by direct sequencing. Figure 1 shows that the codon 12 *ras* mutation harboured by the EJ line is still easily detectable even when the mutant DNA represented only 15% of the sample. Examination of stained sections of all the tumour samples by a trained pathologist revealed that tumour tissue represented at least 45% of the cells in the section and therefore that a mutation would be detected in these cases.

**Figure 1** Sensitivity of PCR and direct sequencing in the detection of mutant Harvey *ras* genes. Normal HEK DNA (GGC) was mixed in different ratios with EJ Bladder carcinoma DNA (GTC) to determine the sensitivity of mutant *ras* detection. The T lane was clearly visible when the EJ DNA represented only 15% of the total DNA.

Table I Benign premalignant and malignant head and neck lesions analysed

| *Lesion* | | *Tested* | *Cultures* | *Treatment prior to surgery* |
|---|---|---|---|---|
| Benign papillomas | | 6 | 2 | 6 $CO_2$ Laser therapy |
| Premalignant erythroplakias | | 5 | 4 | None |
| Malignant SCC | T1 | 4 | 0 | None |
| | T2 | 16 | 1 + 2[a] | 2 DXT[a] |
| | T3 | 12 | 0 | 1 DXT |
| | T4 | 24 | 4 | 1 DXT, Caesium implant |
| Spindle Carcinoma | | 2 | 0 | 2 DXT |
| Lymph node metastases | | 3 | 2 | None |
| *Cell lines* | | | | |
| SCC-4 | | | | DXT, Methotrexate |
| SCC-9 | | | | None |
| SCC-12 Clone B | | | | None |
| SCC-12 Clone F | | | | None |
| SCC-13 | | | | DXT |
| SCC-15 | | | | None |
| SCC-25 | | | | None |

[a]DXT = Deep X-ray Therapy.

*Absence of H-*ras *mutations at all stages of head and neck SCC development*

Table I summarises all the benign, premalignant and SCC biopsies tested for H-*ras* mutations, and in some cases the keratinocytes cultured from them. No *ras* mutations were present in six benign papillomas, five premalignant erythroplakias, 4 T1, 16 T2, 12 T3 and 24 T4 and two spindle cell malignant SCC, all but five of which were untreated. Also, no mutations were detected in three SCC lymph node metastases. In addition cultures of four erythroplakia biopsies and 16 SCC cell lines all possessed normal H-*ras* genes.

### Discussion

We have confirmed and extended the observations of previous investigators (Rumsby *et al.*, 1990; Sheng *et al.*, 1990; Chang *et al.*, 1991) to demonstrate that mutations in the H-*ras* proto-oncogene are extremely rare in the pathogenesis of H & N SCC in Caucasian patients. Extending the findings of others, we were unable to find H-*ras* mutations in 11 premalignant or benign lesions and 20 early stage (T1, T2) untreated SCC which were not studied by the earlier investigators. Similar findings were also reported recently by Yeudall *et al.* (1993) who could not detect *ras* mutations in any dysplastic oral biopsies from three patients. Taken together these results do not support the hypothesis that H-*ras* mutations are present during the early stages of H & N SCC development and are later selected against during tumour progression (see Introduction). The amount of tumour present in each biopsy should have been enough to render a mutation readily detectable by direct sequencing (see Figure 1). Also in several cases cultures of epithelial cells from the tumours or established tumorigenic cell lines were studied (Table I) and these were unlikely to be contaminated with normal cells. It is unlikely therefore that any mutations in the H-*ras* gene would have been missed because of excessive normal cell contamination.

The absence of H−*ras* mutations in Western samples of H & N SCC contrasts with the findings of Saranath *et al.* (1991) who reported finding H-*ras* mutations in 35% of Indian SCC samples collected from patients who had habitually chewed betal quid. These results do suggest therefore that *ras* mutations can give keratinocytes a selective advantage in H & N SCC sites and that they do persist throughout tumour progression (see also Corominas *et al.*, 1989). It is also known that *ras* oncogenes can render human keratinocytes more sensitive to EGF *in vitro* and when grafted onto nude mice can regenerate an epithelium reminiscent of premalignant oral leukoplakia (Henrard *et al.*, 1990). Interestingly, tobacco-related oral malignancies in India are usually preceded by premalignant lesions such as leukoplakia (Daftary, 1990) whereas oral malignancies in Western countries only rarely arise from premalignant lesions (Binnie, 1990).

It has been suggested that the absence of *ras* mutations in Western samples could be due to the different types of tobacco used in India and the UK (Chang *et al.*, 1991) but since most UK H & N SCC are thought to have a tobacco smoking-related aetiology (Stell, 1972), and tobacco smoke contains several carcinogens which are known to activate the H-*ras* gene (IARC, 1986; Quintanilla *et al.*, 1986; Brown *et al.*, 1990), this does not seem a satisfactory explanation. Whilst it has still to be demonstrated that *ras* mutations are an early event in the pathogenesis of Indian SCCs (Saranath *et al.*, 1991), it is possible that the betal quid contains tumour promoters which give the *ras* mutations a continuous selective advantage, analagous to the mouse two-stage epidermal tumorigenesis model (see Clark, 1993 for a review). It is known that the quid is held for long periods of time at the site of tumour induction (Chang *et al.*, 1991; Saranath *et al.*, 1991), and continuous exposure to tumour promoters is essential for their action (Boutwell, 1964).

It is clear that keratinocytes harbouring *ras* mutations can gain a continuous selective advantage during the pathogenesis of oral SCC, if the correct conditions for such selection prevail (Saranath *et al.*, 1991). The exact nature of these conditions remains to be established but an answer should emerge when the molecular mechanisms of oral SCC induction in Caucasian and Indian subjects are understood in more detail.

The authors would like to acknowledge J. Wyke and B. Ozanne for critical review of the manuscript. Professor H. Sewell for advice, the MRC for a research training fellowship to L. Clark and the CRC for additional financial support.

### References

ALITALO, K., KUISMANEN, E., MYLLYLA, R., KIISTALA, U., ASKO-SELGAVAARA, S. & VAHERI, A. (1982). Extracellular matrix proteins of human epidermal keratinocytes and feeder 3T3 cells. *J. Cell Biol.*, **94**, 497–505.

BALMAIN, A., RAMSDEN, M., BOWDEN, G.T. & SMITH, J. (1984). Activation of the mouse cellular Harvey-*ras* gene in chemically induced benign skin papillomas. *Nature*, **307**, 658–660.

BINNIE, W.H. (1990). Low risk areas of the world. In *Risk Markers for Oral Diseases*. Vol 2, Johnson, N.W. (ed.) Cambridge University Press: United Kingdom.

BOS, J.L. (1989). *ras* Oncogenes in human cancer: A review. *Cancer Res.*, **49**, 4682–4689.

BOUTWELL, R.K. (1964). Some biological aspects of skin carcinogenesis. *Prog. Exp. Tumor Res.*, **4**, 207–250.

BROWN, K., BUCHMAN, A. & BALMAIN, A. (1990). Carcinogen-induced mutations in the mouse c-Ha-ras gene provide evidence of multiple pathways for tumour progression. *Proc. Natl Acad. Sci. USA*, **87**, 538-542.

CHANG, S.E., BHATIA, P., JOHNSON, N.W., MORGAN, P.R., MCCORMICK, F., YOUNG, B. & HIORNS, L. (1991). *Ras* mutations in United Kingdom examples of oral malignancies are infrequent. *Int. J. Cancer*, **48**, 409-412.

CLARK, L.J. (1993). Oncogenes and ENT. *Clin. Otolarygol.*, **18**, 4-13.

COROMINAS, M., KAMINO, H., LEON, J. & PELLICER, A. (1989). Oncogene activation in human benign tumors at the skin (keratoacanthomas): Is *Hras* involved in differentiation as well as proliferation? *Proc. Natl Acad. Sci. USA*, **86**, 6372-6376.

COROMINAS, M., LEON, J., KAMINO, H., CRUZ-ALVAREZ, M., NOVICK, S.C. & PELLICER, A. (1991). Oncogene involvement in tumor regression: *H-ras* activation in the rabbit keratoacanthoma model. *Oncogene*, **6**, 645-651.

DAFTARY, D.K. (1990). The situation in high risk areas of the world. In *Risk Markers for Oral Diseases* Vol. 2, Johnson, N.W. (ed.) Cambridge University Press: United Kingdom.

DUBEAU, L., CHANDLER, L.A., GRALOW, J.R., NICHOLS, P.W. & JONES, P.A. (1986). Southern blot analysis of DNA extracted from formalin-fixed pathology specimens. *Cancer Res.*, **46**, 2964-2969.

GIBBS, J.B., SIGAL, I.S. & SCOLNICK, E.M. (1985). Biochemical properties of normal and oncogenic *ras* p21. *Trends in Biochem. Sci.*, **10**, 350-353.

GLICK, A.B., SPORN, M.B. & YUSPA, S.H. (1991). Altered regulation of TGF-$\beta$, and TGF-$\alpha$ in primary keratinocytes and papillomas expressing v-Ha-*ras*. *Mol. Carcinog.*, **4**, 210-219.

GROSS-BELLARD, M., OUDET, P. & CHAMBON, P. (1973). Isolation of high-molecular-weight DNA from mammalian cells. *Eur. J. Biochem.*, **36**, 32-38.

HENRARD, D., THORNLEY, A.T., BROWN, M.L. & RHEINWALD, J.G. (1990). Specific effects of *ras* oncogene expression on the growth and histogenesis of human epidermal keratinocytes. *Oncogene*, **5**, 475-481.

HULTMAN, T., BERGH, S., MOKS, T. & UHLEN, M. (1991). Bidirectional solid phase sequencing of *in vitro* amplified plasmid DNA. *Biotechniques*, **10**, 84-93.

IARC (1986). Monographs on the evaluation of the carcinogenic risk of chemicals to humans. Vol **38**. *Tobacco Smoking*, IARC: Lyon.

LEON, J., KAMINO, H., STEINBERG, J.J. & PELLICER, A. (1988). H-*ras* activation in benign and self regressing skin tumors (keratoacanthomas) in both humans and an animal model system. *Mol. Cell Biol.*, **8**, 786-793.

OZANNE, B., FULTON, R.J. & KAPLAN, P.L. (1980). Kirsten murine sarcoma virus-transformed cell lines and a spontaneously transformed rat cell line produce transforming factors. *J. Cell Physiol.*, **105**, 163-180.

PARASKEVA, C., BUCKLE, B.G., SHEER, D. & WIGLEY, C.B. (1984). The isolation and characterization of colorectal epithelial cell lines at different stages in malignant transformation from familial polyposis coli patients. *Int. J. Cancer*, **34**, 49-56.

PARKINSON, E.K. & NEWBOLD, R.F. (1980). Benzo (a) pyrene metabolism and DNA adduct formation in serially cultivated strains of human epidermal keratinocytes. *Int. J. Cancer*, **26**, 289-299.

PATERSON, H., REEVES, B., BROWN, R., HALL, A., FURTH, M., BOS, J., JONES, P. & MARSHALL, C.J. (1987). Activated N-*ras* controls the transformed phenotype of HT1080 human fibrosarcoma cells. *Cell*, **51**, 803-812.

QUINTANILLA, M., BROWN, K., RAMSDEN, M. & BALMAIN, A. (1986). Carcinogen-specific mutation and amplification of Ha-*ras* during mouse skin carcinogenesis. *Nature*, **322**, 78-80.

RHEINWALD, J.G. & BECKETT, M.A. (1981). Tumorigenic keratinocyte lines requiring anchorage and fibroblast support cultured from human squamous cell carcinomas. *Cancer Res.*, **41**, 1657-1663.

RUMSBY, G., CARTER, R.L. & GUSTERSON, B.A. (1990). Low incidence of *ras* oncogene activation in human squamous cell carcinomas. *Br. J. Cancer*, **61**, 365-368.

SANKARANARAYANAN, R. (1990). Oral Cancer in India: An epidemiologic and clinical review. *Oral Surg. Oral Med. Oral Pathol.*, **69**, 325-330.

SARANATH, D., CHANG, S.E., BHOITE, L.T., PANCKAL, R.G., KERR, I.B., MEHTA, A.R., JOHNSON, N.W. & DEO, M.G. (1991). High frequency mutation in codons 12 and 61 of H-*ras* oncogenes in chewing tobacco related human oral cancer in India. *Br. J. Cancer*, **63**, 573-578.

SHENG, Z.M., BARROIS, M., KLIJANIENKO, J., MICHEAU, C., RICHARD, J.M. & RIOU, G. (1990). Analysis of the c-Ha-*ras*-1 gene for deletion, mutation, amplification and expression in lymph node metastases of human head and neck carcinomas. *Br. J. Cancer*, **62**, 398-404.

STELL, P.M. (1972). Smoking and laryngeal cancer. *Lancet*, **i**, 617-618.

TABIN, C.J., BRADLEY, S.M., BARGMANN, C.I., WEINBERG, R.A., PAPAGEORGE, A.G., SCOLNICK, E.M., DUAR, R., LOWY, D.R. & CHANG, E.H. (1982). Mechanism of activation of a human oncogene. *Nature*, **300**, 143-149.

UICC (1987). *Union Internationale Contre Le Cancer TNM Classification of Malignant Tumours*. Hermanek, P. & Fobin, L. (eds) Springer-Verlag: Heidelberg.

WINTON, D.J., BLOUNT, M.A. & PONDER, B.A.J. (1989). Polyclonal origin of mouse skin papillomas. *Br. J. Cancer*, **60**, 59-63.

YEUDALL, W.A., TORRANCE, L.K., ELSEGOOD, K.A., SPEIGHT, P., SCULLY, C. & PRIME, S.S. (1993). *Ras* gene point mutation is a rare event in premalignant tissues and malignant cells and tissues from oral mucosal lesions. *Oral Oncol.* Eur. J. Cancer, **29B**, 63-67.